# Effects of Youth Tobacco Access and Possession Policy Interventions on Heavy Adolescent Smokers

**DOI:** 10.3390/ijerph6010001

**Published:** 2008-12-23

**Authors:** Leonard A. Jason, Steven B. Pokorny, Monica L. Adams, Annie Topliff, Courtney C. Harris, Yvonne Hunt

**Affiliations:** 1 DePaul University, Center for Community Research, 990 Fullerton Avenue, Suite 3100, Chicago, Illinois 60614, USA; E-mails: madams8@depaul.edu (M. L. A.); atopliff@gmail.com (A. T.); charri30@depaul.edu (C. C. H.); 2 University of Florida, Department of Health Education & Behavior, P.O. Box 118210 / FLG - 5 Gainesville, FL 32611-8210, USA; E-mail: spokorny@hhp.ufl.edu (S. B. P.); 3 National Cancer Institute, 6116 Executive Boulevard, Room 3036A, Bethesda, MD 20892-8322, USA; E-mail: yvonnehunt@gmail.com (Y. H.)

**Keywords:** PUP laws, minors’ access to tobacco

## Abstract

This study evaluated the effects of tobacco PUP (Purchase, Use and Possession) laws on tobacco use patterns among students in twenty-four towns, which were randomly assigned into an experimental and a control group. The experimental group involved both PUP law enforcement and reducing minors’ access to commercial sources of tobacco, and the condition for the control group involved only efforts to reduce minors’ access to commercial sources of tobacco. The present study found that adolescents in the control group had a significantly greater increase in the percentage of youth who smoked 20 or more cigarettes per day when compared to the experimental group.

## 1. Introduction

Several studies have suggested that tobacco Possession-Use-Purchase Laws (PUP) help reduce youth tobacco use [1–5]. In an eight-town randomized study, white youths who lived in communities with strict enforcement of tobacco sales and possession laws had significantly fewer increases in the prevalence of tobacco use over time than those living in communities with only moderate enforcement of tobacco sales laws [6]. Wakefield and Giovino [7] have correctly noted that small sample sizes, non-randomized designs, and lack of long term follow-up have weakened conclusions that might be made about most published studies in this area.

One of the more recent randomized studies in this area was completed by Jason *et al*. [8], who randomly grouped students from 24 towns based on two conditions. One intervention, called the experimental condition, involved both fining minors for possession of tobacco (PUP law enforcement) and reducing commercial sources of access to tobacco. The second intervention, called a control condition, focused only on efforts to reduce commercial sources of access to tobacco. Junior high and high school students in 24 towns were surveyed. Findings indicated that rates of current smoking increased at a significantly slower rate for adolescents in towns where PUP laws were enforced. Current smoking for students in the control condition increased 4.1% (from 8.9% to 13.0%) versus just 1.9% (from 9.0% to 10.9%) for those in the experimental condition. While this result is modest, the public health implications are still potentially important. In addition, Jason, Hunt, Adams, Pokorny, and Gadiraju [9] recently reported that 5 out of 12 experimental condition communities (42%) adopted local 100% smoke-free ordinances after our research team had worked with them to increase PUP law enforcement; whereas only 1 of the 12 (8%) control communities that had not worked with us to increase PUP law enforcement adopted 100% smoke-free ordinances. It is possible that these types of PUP laws might also affect other behaviors such as heavy use of tobacco.

Pokorny, Jason, and Schoeny [10] found that retail availability of tobacco use was related to initiation of tobacco use, but not continued tobacco use. In other words, these authors found that making it more difficult to purchase tobacco was more effective for those who were experimenting with tobacco use, whereas those who were more addicted would find other ways of obtaining their tobacco when commercial sources were decreased. These authors suggested that those youth who are more addicted to tobacco might need stronger anti-tobacco policies than just reducing retail availability in order to impact their tobacco use. It is possible that PUP laws might be needed to reduce smoking among more addicted heavier tobacco users.

There is a growing literature on youth who are heavy tobacco users. For example, Cornelius *et al.* [11] found two thirds of current adolescent smokers indicated that they were heavy smokers. Sussman [12] found that adolescents who consume cigarettes regularly are at greater risk for using other drugs. In addition, Henningfield, Michaelides, and Sussman [13] found that adolescents’ cessation of smoking is related to their daily consumption of cigarettes. Those who smoked more cigarettes were less likely to quit. It is clear that more research needs to focus on how to help adolescents who are heavy smokers quit using tobacco.

The present study used the data set described in Jason *et al.*’s [8] randomized study of 24 towns to investigate patterns of tobacco use among heavier students. It was hypothesized that towns exposed to an intervention designed to strengthen enforcement of PUP laws would result in less students smoking 20 or more cigarettes per day, compared to towns that did not actively increase their PUP enforcement efforts.

## 2. Methods

### 2.1. Procedures

The Youth Tobacco Access Project involved 24 towns in Illinois, with four cohorts of data collected from these towns in the spring of 2002, 2003, 2004, and 2005 (see Jason *et al*. [8] for more details about town selection). In 2001, the 24 selected towns were matched for population size and median income and then randomly assigned to the two conditions. The control (C) condition involved efforts to reduce commercial sources of youth access to tobacco, and the Experimental (E) condition involved efforts to both reduce commercial sources of youth access to tobacco and fine minors for possessing or using tobacco. Minors received a civic fine for tobacco PUP law violations (approximately $75). Police officers were instructed to issue citations to minors who were caught possessing tobacco in public locations. In most towns, we worked with one or two police officers to ensure that they implemented these procedures.

The C and E towns did not differ significantly at baseline on population size, median household income, and commercial illegal sales of tobacco to minors. In all communities, we wanted each town to have less than 20% illegal commercial sales of cigarettes to minors (based on recommendations in the US from the Synar amendment). We only worked with towns that had contracts with the Illinois Liquor Control Commission, and with those that had agreed to have their police do three yearly enforcements of all merchants selling tobacco products. In other words, all towns were participating in the supply side of tobacco control activities, with regular merchant enforcements to reduce illegal sales of tobacco. Overall rates of illegal sales between the E and C conditions did not differ over the intervention. Merchants in all Illinois towns are prohibited from selling tobacco products to minors under the age of 18. Stores that sell cigarettes are also required to post signs, indicating the law against selling cigarettes to minors.

The 12 E communities agreed to initiate or increase PUP law enforcement practices, whereas the 12 C communities received instructions to maintain their current low levels of PUP law enforcement. There were no significant E versus C differences at baseline in PUP law citations. Over a four year period, the average yearly number of PUP law citations issued to minors within the E communities was significantly higher than those within the C communities (*t*(22) = –2.30, *p* = .03), indicating that PUP enforcement was, in fact, stronger in E (M = 16.54) than C towns (M = 6.31).

### 2.2. Student Participants

Our survey was administered to students in grades seven to ten during 2002, grades seven to eleven in 2003, and grades seven to twelve in 2004 and 2005 (see Jason *et al*. [8] for more details about survey administration). Across the four waves of data collection for the present study, a total of 52,550 students were eligible to be surveyed (i.e., enrolled in a target grade at a participating school) in one or more waves. In 11 of the 41 participating schools, school administrators selected only students who lived in the target towns to be eligible for surveys. Of the eligible students, parental consent forms were obtained for 33,991 (65%) students. A total of 29,851 eligible students (57%) completed the survey during at least one wave of data collection. Over the course of four waves, a total of 59,160 surveys were completed, representing an average of two waves of data for each participating student. Of the 59,160 surveys, 482 (0.8%) were excluded from the analyses because of inconsistent or invalid responding across survey items. Because the analyses included a town-level covariate, 7,953 (13%) surveys (i.e., from 4,630 students) were excluded from analyses because the students lived outside of the participating towns and, therefore, were not directly exposed to the intervention. The final sample for the present analyses included 25,404 students and 50,725 assessments.

### 2.3. Measures

#### Student Survey

The Youth Tobacco Access Project’s Student Survey is a 74 item self-report survey developed to assess students’ demographic variables (i.e., gender, race, grade), as well as their attitudes and behaviors toward tobacco and other drugs [6, 14, 15].

#### Level-1 Variables

All Level-1 variables were derived from self-report data obtained from the student survey. Only variables expected to change from wave to wave were selected as level-1 time-varying covariates (i.e., friends who use tobacco).

*Outcome variables*. For the purposes of the current analyses, we asked the question: “On the days that you usually smoke cigarettes, how many do you usually smoke”, and evaluated whether or not a student used 20 or more cigarettes per day, as an indicator of a more addicted tobacco user.

*Time*. Time was modeled as a Level-1 variable and represents the 4 waves of assessment.

*Closest Friend tobacco users*. The presence of closest friend tobacco users in the student’s life was calculated as a continuous variable based on the response to the question: “How many of your four closest friends use tobacco? (None, 1, 2, 3, and 4).”

#### Level-2 Variables

All Level-2 variables represent stable student-level characteristics and were also derived from self-report data obtained from the student survey.

*Grade.* Grade was determined from the grade the student was in at the start of the study in 2002. Grade was grand mean centered.

*Race.* Race was determined from responses to the questions “Are you Latino or Hispanic origin?” (Yes or No) and “How do you describe yourself? Mark all that apply: Asian, Black/African American, Middle Eastern, Native American/Alaskan Native, Native Hawaiian/Other Pacific Islander, White/Caucasian, Other.” Because the majority of students were White, African American, or Latino, this variable was reduced to four categories (i.e., White, African American, Latino, and Other). For the present analyses, this variable was indicator (i.e., dummy) coded by creating dichotomous variables, indicating African American, Latino, and Other. Therefore, in all analyses, White youth were the reference group for each of the three dummy coded variables.

*Gender.* Gender was coded as a dichotomous variable determined from responses to the question: “What is your gender? (Female or Male).” Females were coded as 0 and males as 1.

*Adult tobacco users.* The presence of an adult tobacco user in the home was calculated as a dichotomous variable determined by the response to the question: “Is there an adult (someone over 18 years old) living in your home who uses tobacco? (Yes or No).” No was scored as 0 and yes as 1.

#### Level-3 Variables

The Level-3 variables represent community-level constructs.

*Experimental versus Control condition.* The 12 towns randomly assigned to receive support to increase PUP law citations were in the E condition (with a score of 1), whereas the 12 towns randomly assigned to receive no support for increasing PUP law enforcement and consequently having lower levels of PUP law citations were in the C condition (with a score of 0).

*Proportion of Commercial Tobacco Sales to Youth.* Two assessments of the proportion of commercial tobacco sales to youth occurred; one was at Year 2 and the other at Year 4. The average of the two assessments carried out by our DePaul University staff represents the proportion of commercial tobacco sales to youth, which is the variable used in our HLM analysis. We used commercial sales data from our reports, rather than those from the police enforcements, as we had more control over these procedures.

*Household Income.* The median Household Income in thousands of dollars for each town was coded as a continuous variable based on the 2000 Census data. This variable was grand mean centered (*M* = $59,726; *SD* = $20,785) to represent the mean household income across the towns.

### 2.4. Statistical Analysis

A random coefficient multilevel analysis was performed using HLM 6.03 [16]. This analytical approach was selected due to the multilevel data (i.e., observations within individuals within towns). Because the outcome was dichotomous (i.e., whether or not someone was currently smoking 20 or more cigarettes per day), a Bernoulli model was selected, which specifies a binomial distribution and a logit-link function. Our interpretation focused on the population-average model as it tests for an intervention effect averaging across towns.

Because closest friends who use tobacco might change over time, we placed friends as a level-1, time-varying covariate. At levels-2 and -3, the intercept was allowed to randomly vary, accounting for random variability in the outcome measures across individuals and towns. The wave slope was also modeled as random at level-2, based on our prediction that individuals would vary in the likelihood that they would smoke over time. At level-2 (i.e., person-level), we included grade, race, gender, and adult tobacco users as covariates. At level-3 (i.e., town-level), we included experimental condition, town household income, and commercial availability of tobacco to minors.

## 3. Results

The E and C towns did not differ significantly at baseline on PUP law violations, population size, median household income, commercial illegal sales of tobacco to minors, gender, race, grade level, the presence of adult smokers in the home, or friends who use tobacco. For the dependent variable, results from an unconditional model revealed significant between-town variation in current drug use (*p* < .01). In the next step, all student-level (i.e., level-2) and the town-level (i.e., level-3) variables were added to the model.

### 3.1. Smoking 20 or More Cigarettes per Day

Controlling for a variety of individual-level variables (described below), the odds that students smoked 20 or more cigarettes per day at the start of the study was 0.002; 95% *CI .001* – .002, an effect that did not vary by treatment condition. At baseline, a number of individual factors significantly increased the likelihood of students using 20 or more cigarettes per day: number of friends who used tobacco (*OR* = 2.30; 95% *CI* 2.18 – 2.44), students who belonged to higher grade levels (*OR* = 1.07; 95% *CI* 1.00 – 1.13), and whether a student was male (*OR* = 3.44; 95% *CI* 2.84 – 4.16).

**Figure 1.**
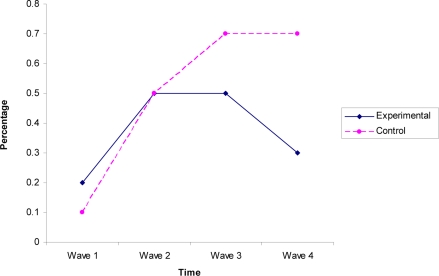
Participants Smoking 20 or More Cigarettes per Day.

Over time, the E treatment condition at the town level in comparison to the C condition was significantly associated with lower likelihood of students smoking 20 or more cigarettes per day (*OR* = .79; 95% *CI* .67 – .94). This meant that the slopes for the E and C condition were significantly different over Waves 1 through 4 (See Figure 1).

## 4. Discussion

The present study found that the change in the likelihood of youth smoking 20 or more cigarettes per day differed over time, which showed greater increases in the adolescents in control towns (increasing from Wave 1 to Wave 4 from .1% to .7%) than in youth from the experimental towns (increasing from Wave 1 to Wave 4 from .2% to .3%). These results complement the findings by Jason *et al.* [8], who found differences in student use of current smoking. These studies support the efficacy of combined approaches involving efforts to both reduce youth access to tobacco as well as provide consequences for adolescent use of tobacco. This current research suggests that the enforcement of PUP laws does impact youth who smoke 20 or more cigarettes daily.

At the start of the study, there were a number of variables that were related to heavy smoking among youth. At baseline, it was understandable that heavy smokers were more likely to be from higher grades, have more friends who used tobacco, and tended to be male. In a sense, children who are older and associate more with other smokers are more likely to be heavier smokers, and this appears to be more common among boys than girls.

From a public health standpoint, rather than completely suppressing teen smoking, it might be more important to reduce the visibility of youth smoking in public, which may minimize the effects of modeling. Possession bans make it easier to reduce subtle peer pressure when students congregate at social events and publicly smoke and encourage others to engage in this behavior. High levels of visible tobacco use by minors might also lead them to overestimate the actual proportion of children who use tobacco [17]. Subsequently, children may feel pressure to conform to what they incorrectly perceive as the norm [18]. Leventhal, Fleming, and Glynn [19] found that youth overestimated the proportion of peers and adults who smoked by at least a factor of three, and Alesci, Forster, and Blaine [20] found that youth who witnessed youth or adult smoking in various public locations were more likely to perceive smoking as a socially acceptable behavior. Therefore, reducing youth smoking in public may play a key role in reducing youth smoking rates by reducing the perception of smoking as a normal and acceptable behavior.

There is considerable controversy regarding the appropriateness of tobacco PUP laws and specific concerns about the effects of these laws on individuals who violate them. Some anti-smoking coalitions are opposed to these measures because they believe these laws might make youth the offenders rather than the victims of the tobacco industry’s efforts to recruit new smokers. These types of coalitions also believe that by shifting enforcement efforts to teenagers, the real offenders who sell these deadly products to minors are protected from being fined. While focusing just on fining youth for possession of tobacco is inappropriate, a combined approach involving consequences for both merchants who illegally sell tobacco and youth who illegally possess tobacco is appropriate. There is a clear need to investigate this issue further and determine whether these policies might influence rates of smoking among youth.

Some public health advocates have been uncomfortable with the use of punishment, which they define as an aversive or unpleasant event that suppresses or prevents the occurrence of a behavior [7]. However, punishment can also be defined as any consequence of behavior that reduces the future occurrence of that behavior, suggesting that these consequences need not be explicitly unpleasant or aversive. For example, a PUP violation could result in the youth entering a cessation program. Milton *et al*. [21] found that one state that passed PUP laws created a demand for youth cessation interventions. This may be one way of involving the parents without alienating the children. Parent involvement in these efforts might be a critical factor in a program’s success. Many traditional youth cessation programs choose not to notify the parents under the assumption that children do not want their parents to know that they smoke.

Wakefield and Giovino [7] also argue that even if these laws could be enforced at such a level, it is unlikely that police departments would have the additional resources necessary to do so without diverting resources from other enforcement activities. However, additional resources may not be necessary to enforce PUP laws. For example, we have found that police officers are able to enforce possession laws in conjunction with their normal duties, like enforcement of curfew and traffic laws, which requires no additional resources.

There are several limitations in this study. Because we needed to obtain active consent, we were only able to recruit about 50% of the available youth. Additionally, losses to follow-up were high and we did not obtain any biochemical confirmation of self-reported tobacco abstinence. It is unclear what the longer term influence of PUP laws on tobacco use might be after youth finish high school. Finally, there is the possibility that youth might be less likely to report smoking or may report using fewer cigarettes per day if they perceive this as behavior that is punishable. In other words, youth who are surveyed in a school environment may be more likely not to report behavior they can be punished for.

Clearly, there are a number of comprehensive community-based interventions that have also been effectively implemented in the United States [22]. Cummings [23] states that the most effective demand-reducing influences on tobacco use involve increasing the cost of using tobacco products, comprehensive advertising bans, paid counter-advertising, and smoke-free policies. Among the different environmental strategies, reducing youth access to tobacco products and fines for possession represent important public health strategies to address the problem of youth tobacco use.
